# Genome-Wide Association Analyses Point to Candidate Genes for Electric Shock Avoidance in *Drosophila melanogaster*

**DOI:** 10.1371/journal.pone.0126986

**Published:** 2015-05-18

**Authors:** Mirjam Appel, Claus-Jürgen Scholz, Tobias Müller, Marcus Dittrich, Christian König, Marie Bockstaller, Tuba Oguz, Afshin Khalili, Emmanuel Antwi-Adjei, Tamas Schauer, Carla Margulies, Hiromu Tanimoto, Ayse Yarali

**Affiliations:** 1 Research Group Molecular Systems Biology of Learning, Leibniz Institute of Neurobiology, Magdeburg, Germany; 2 Max Planck Institute of Neurobiology, Martinsried, Germany; 3 Laboratory for Microarray Applications, IZKF, University of Würzburg, Würzburg, Germany; 4 Department of Bioinformatics, Biocenter, University of Würzburg, Würzburg, Germany; 5 Institute of Human Genetics, University of Würzburg, Würzburg, Germany; 6 Department of Physiological Chemistry, Butenandt Institute and LMU Biomedical Center, Faculty of Medicine, Ludwig-Maximilians-University of Munich, Munich, Germany; 7 Tohoku University Graduate School of Life Sciences, Sendai, Japan; 8 Center for Behavioral Brain Sciences, Magdeburg, Germany; Alexander Fleming Biomedical Sciences Research Center, GREECE

## Abstract

Electric shock is a common stimulus for nociception-research and the most widely used reinforcement in aversive associative learning experiments. Yet, nothing is known about the mechanisms it recruits at the periphery. To help fill this gap, we undertook a genome-wide association analysis using 38 inbred *Drosophila melanogaster* strains, which avoided shock to varying extents. We identified 514 genes whose expression levels and/ or sequences co-varied with shock avoidance scores. We independently scrutinized 14 of these genes using mutants, validating the effect of 7 of them on shock avoidance. This emphasizes the value of our candidate gene list as a guide for follow-up research. In addition, by integrating our association results with external protein-protein interaction data we obtained a shock avoidance-associated network of 38 genes. Both this network and the original candidate list contained a substantial number of genes that affect mechanosensory bristles, which are hair-like organs distributed across the fly’s body. These results may point to a potential role for mechanosensory bristles in shock sensation. Thus, we not only provide a first list of candidate genes for shock avoidance, but also point to an interesting new hypothesis on nociceptive mechanisms.

## Introduction

Electric shock induces strong defensive and aversive behaviour in animals and is rated as painful by humans. Accordingly, it has become a traditional aversive reinforcement for associative learning research across species, including humans, e.g., [1–6]. Although the neurons that mediate the reinforcing effect of shock are fairly well-studied, e.g., [7,8], nothing is known about the processes recruited by electric shock at the sensory periphery. To help fill this gap, we took a genome-wide approach in the fruit fly *Drosophila melanogaster*, which has well-known advantages when it comes to detecting gene-behaviour relationships, which are often conserved through evolution [9–11].

Instead of using artificial fly mutants, we took advantage of natural variation. We tested 38 nature-derived inbred fly strains in shock avoidance and then looked for associations between their behavioural scores and both gene expression level- and single nucleotide polymorphism (SNP)-data, which were already available [12,13]. This strategy, which has been successfully applied to a variety of behavioural traits, e.g., [14–17] differs from canonical mutagenesis screens in that it probes for the individually small effects of a multitude of genes all at once, instead of looking for more obvious consequences of mutations on a gene-by-gene basis. This approach is likely to provide a more realistic picture of the quantitative variation in behaviour and the underlying genetic bases [18,19].

With this strategy, we uncovered 514 candidate genes for shock avoidance. We independently scrutinized 14 of these genes using mutants and found shock avoidance-roles for 7 of them, corroborating the validity of our genome-wide approach. In addition, we integrated our association analysis results with existing protein-protein interaction data, revealing a shock avoidance-associated network of 38 genes. This network, as well as the original candidate list contained a substantial number of genes relevant for mechanosensory bristles—hair-like body-surface organs, potentially suggesting a role for these in the sensation of shock.

## Results and Discussion

### Genome-wide association analyses for electric shock avoidance

**Fig 1:**
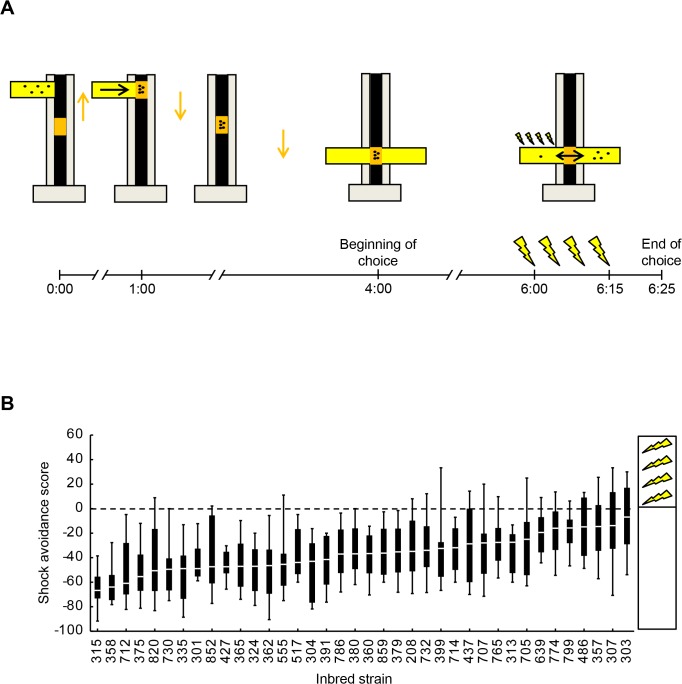
Shock avoidance of 38 inbred *Drosophila melanogaster* strains. A. For the shock avoidance assay, flies (represented by black dots) were loaded into the setup using ‘shock tubes’ (coloured yellow) at 0:00 min. At 1:00 min, they were transferred to a movable ‘mid-compartment’ (coloured orange). At 4:00 min, the mid-compartment was moved to the choice point of a maze with shock tubes as the two arms. After 2-min dispersal time, one of the maze-arms was applied with four pulses of electric shock (represented by yellow lightening symbols). 10 s after the last pulse, the maze-arms were sealed and the flies in each arm were counted to calculate a shock avoidance score. Negative values indicated avoidance of the shocked maze-arm. Orange and black arrows represent the movement of the mid-compartment and of the flies, respectively. B. The 38 tested inbred strains had significantly different shock avoidance scores. Box plots show the median as the midline, 25 and 75% as the box boundaries and 10 and 90% as the whiskers. Sample sizes were from left to right N = 32, 16, 22, 24, 24, 16, 16, 24, 26, 28, 16, 24, 28, 34, 16, 32, 24, 22, 18, 18, 22, 32, 16, 16, 24, 16, 28, 18, 22, 16, 30, 16, 20, 24, 16, 20, 32, 24.

We tested 38 wild-derived inbred fruit fly strains for their choice in a maze with one electrified and one non-electrified arm (Fig 1A). The resulting shock avoidance scores significantly differed across the strains (Fig 1B: Kruskal-Wallis test: H = 158.57, d.f. = 37 and *P*< 0.0001; for sex-specific scores and analyses, see S1 Fig). Critically, this variation in shock avoidance is unlikely to be a simple consequence of activity level, as we found no evidence for a correlation between the present median shock avoidance scores and the mean scores for locomotor activity upon mechanical disturbance as measured by Ayroles et al. [12] (Pearson correlation: Females: R^2^ = 0.0002, *P* = 0.94; Males: R^2^ = 0.0080, *P* = 0.59, N = 38 in each case).

**Fig 2:**
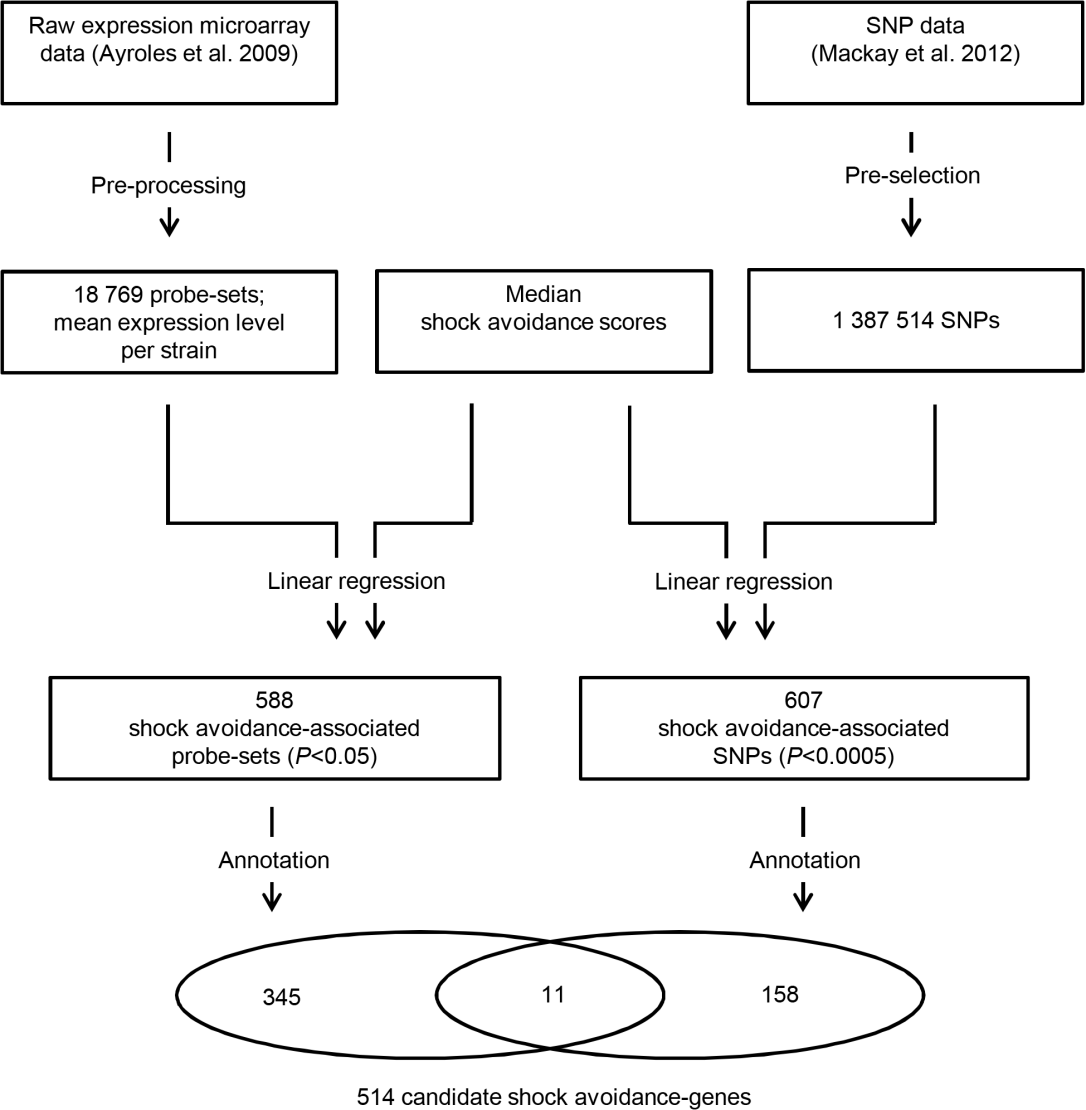
Genome-wide association analyses for shock avoidance. On the left, the gene expression level—shock avoidance association analysis is sketched. After pre-processing the raw expression microarray data, for each of the 18 769 probe-sets, we tested for a linear relationship across strains between the mean expression levels and the median shock avoidance scores. This analysis suggested 588 shock avoidance-associated probe-sets (linear regression *P*< 0.05; see S1 Table for a list with full statistical reports), corresponding to 356 candidate genes (see S4 Table for a list). On the right, the single nucleotide polymorphism (SNP)—shock avoidance association analysis is shown. We narrowed down our analysis to pre-selected SNPs with favourable minor allele frequencies and call rates. Testing for relationships between the allele types and the shock avoidance scores suggested 607 shock avoidance-associated SNPs (linear regression *P*< 0.0005; see S2 and S3 Tables for a list with full statistical reports), pointing to 169 candidate shock avoidance-genes (see S4 Table for a list), 11 of which were already suggested by the expression level-associations.

The inbred strains used had already been characterized in terms of their naturally varying gene expression levels as well as genome sequences [12,13]. We probed for associations between these data and the variation in shock avoidance. To relate gene expression levels to shock avoidance (Fig 2, left), we used the raw expression microarray data provided by Ayroles et al. [12]. For each of the 18 769 probe-sets, we tested for an effect of the mean expression level on the median shock avoidance score. None of the probe-sets would have given a statistically significant association using a strict threshold taking into account multiple testing (e.g., *P*< 0.05/ 18 769, corresponding to a Bonferroni correction). We thus refrained from a family-wise error-rate calculation and considered the 588 cases with *P*< 0.05 to be suggestive associations (see S1 Table for a list of these probe-sets along with full statistical reports), pointing to 356 candidate genes (see S4 Table for a list of these genes).

To relate sequence polymorphisms to shock avoidance (Fig 2, right), we relied on the single nucleotide polymorphisms (SNP)-data from Mackay et al. [13]. For each bi-allelic SNP, whose different alleles were well-represented across the 38 inbred strains, we tested for an effect of the allele-type on the median shock avoidance score using linear regression. Again, we would have found no significant associations had we used a Bonferroni-corrected statistical threshold (i.e., *P*< 0.05/ 1 387 514). We considered the 607 cases with *P*< 0.0005 to be suggestive associations (see S2 and S3 Tables for lists of these SNPs along with full statistical reports), pointing to 169 candidate genes, 11 of which were also suggested by the gene expression associations (see S4 Table for a list of candidate genes).

Thus, suggestive gene expression- and SNP-associations with shock avoidance scores pointed to a total of 514 candidate genes (S4 Table), given the particular statistical thresholds mentioned above. These genes may encode for proteins with developmental or acute functions in steps ranging from peripheral sensation of shock down to the muscle contractions for avoidance, thus providing hypotheses with respect to these processes. In support of this, we found Gene Ontology Terms related to, e.g., neuronal development and to a lesser extent to locomotion to be enriched amongst our candidate genes (see S5 Table for a detailed account). In addition, 15 of the candidate genes had been identified as nociception-relevant in an RNAi-based screen for heat avoidance [20] (marked in S4 Table); also *amn* and *dnc* have reported roles in nociceptive behaviour [21] (marked in S4 Table), raising the question whether electric shock, an unnatural stimulus for most animals, owes its potent effect to activation of peripheral receptors evolved for other, natural nociceptive stimuli (for a comparison of reinforcement-signalling of shock *vs*. heat, see [22]). Furthermore, 8 of our candidate genes turned out to be relevant for shock-reinforced olfactory associative learning (marked in S4 Table, see the references therein); we cannot at present distinguish whether they are critical for peripheral detection of shock upstream of the aversive reinforcement pathway or whether their roles in reflexive shock responsiveness and shock-reinforced learning are independent from each other.

### A shock avoidance-associated gene network

**Fig 3:**
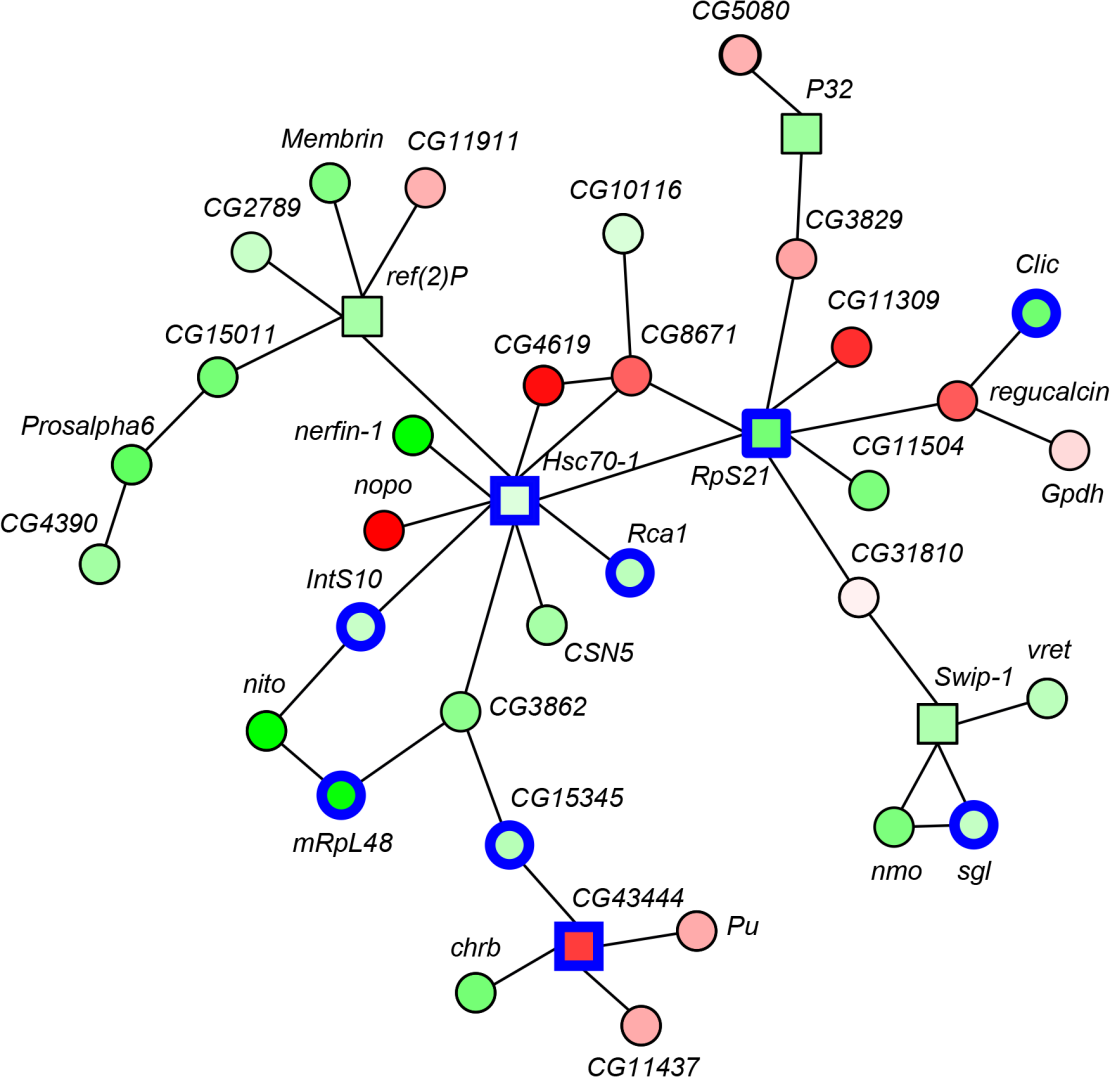
A shock avoidance-associated gene network. Each of the 38 nodes in the network represents a gene associated with shock avoidance in terms of expression level (see S6 Table for a list). Each edge indicates a pair-wise physical interaction between the proteins encoded by the respective genes, based on independent empirical evidence. Shades of green mean that the higher the respective gene’s expression level, the stronger the shock avoidance was. Shades of red mean the converse, i.e., the higher the expression level the weaker the shock avoidance. The darker the shading, the greater the estimated effect of expression level on shock avoidance was. Circles represent genes with a statistically strong association with shock avoidance resulting in a positive network score. Potential functionally related genes with less significant association (negative scores; represented by squares) were included to form connections between the more strongly associated genes. Genes implicated in bristle-function are haloed blue.

In order to explore the interactions between our candidate genes, we made use of a large, experimental evidence-based network featuring 5 280 genes and 63 796 pair-wise physical interactions between the encoded proteins (www.flybase.org) [23]. We assigned a score to each gene in this network on the basis of the statistical reliability of the expression level—shock avoidance association as reported in S1 Table. The optimally scoring sub-network was then computed [24], revealing a smaller, shock avoidance-associated network of 38 genes (Fig 3 and S6 Table).

Two emergent properties were notable. First, genes with a weak association with shock avoidance constituted the central nodes (Fig 3, squares) and were surrounded by the genes with strong association with shock avoidance (Fig 3, circles). The well-connectedness of these central node-genes may point to the higher pleiotropy of their roles, which may have kept them more ‘static’ in the natural population as their variation would be too costly. This would explain the weakness of the shock avoidance associations of these genes, as our analyses relied on the variability of the respective expression levels across the inbred strains. Second, from the 38 network genes, for only 24, at least one phenotypic effect of genetic interference was reported (www.flybase.org) [23]. Of these 24, 9 turned out to be implicated in the function of hair-like body-surface mechanosensory organs, called ‘bristles’ (Fig 3, blue haloes) (marked in S6 Table, see the references therein). These 9 genes mostly clustered; thus, other genes with several bristle-related interaction partners (e.g., *nito*, *CG3862*, *regucalcin*, *CG8671*) may now be attractive candidates for bristle-relevant function. Thus, although we cannot exclude the possibility that other, bristle-independent cellular functions of these genes may make them critical for shock avoidance, it is tempting to hypothesize a potential role for mechanosensory bristles in the sensation of shock. In support of this, among our 514 candidate shock avoidance genes, 91 were identified in mutagenesis and/ or RNAi screens as relevant for mechanosensory bristles (marked in S4 Table, see the references therein).

### Independent validation of candidate genes

As in any other genome-wide association study, the present study must likewise take into account the possible inter-dependency of alleles at different polymorphic loci (i.e. linkage disequilibrium) and, similarly, the inter-correlations between expression levels of different genes [12]. In other words, some candidate genes, although associated with shock avoidance, will not be causally related to it; causal relationships will need to be validated using reverse genetic methods. Such independent validation seemed especially warranted, given our relatively non-stringent statistical thresholds for candidateship.

**Fig 4:**
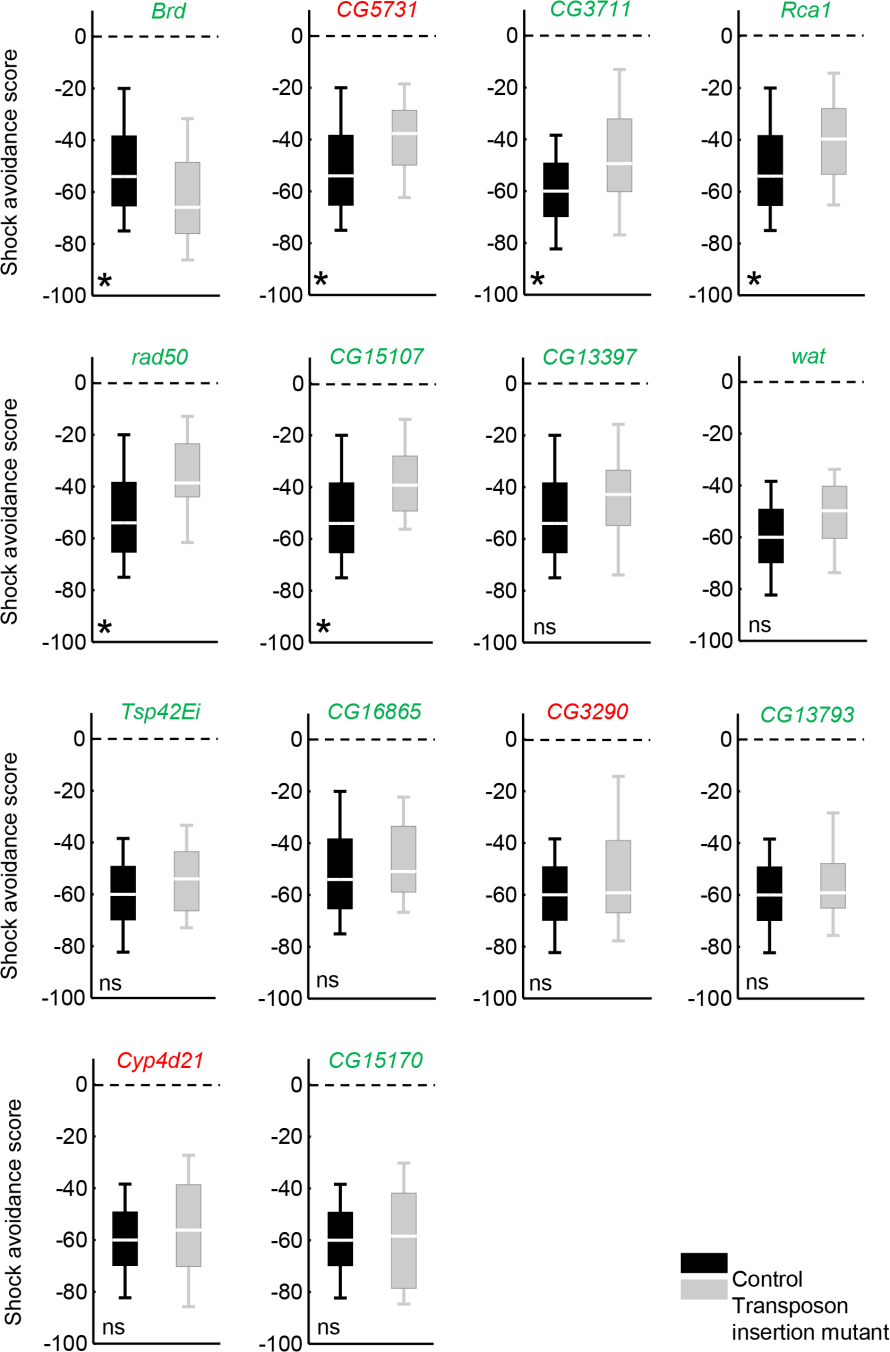
Independent validation of candidate shock avoidance genes using transposon insertion mutants. Each panel shows, for a selected candidate gene, the shock avoidance scores of a respective transposon insertion mutant *vs*. those of the corresponding control (see S7 Table for full genotypes). The colour of the font indicates the direction of the gene expression level—shock avoidance association (i.e. green: the higher the expression level, the stronger the shock avoidance; red: the higher the expression level, the weaker the shock avoidance). In 6 out of 14 cases, shock avoidance scores significantly differed between the genotypes. *: FDR< 0.05, ns: FDR≥ 0.05. Sample sizes are given in S7 Table. Box plots are as explained in Fig 1B.

**Fig 5:**
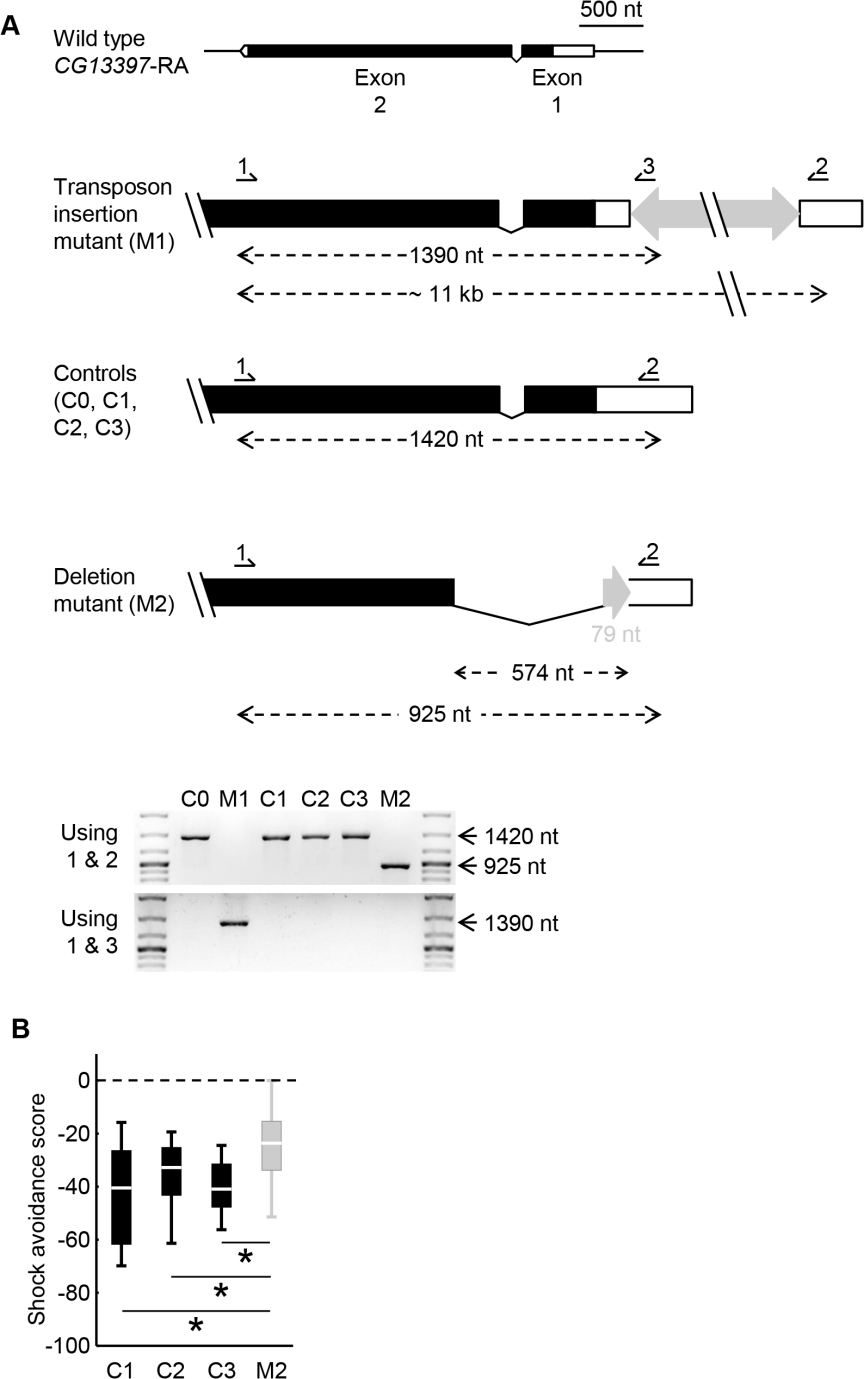
Validation of candidate shock avoidance gene *CG13397* using a deletion mutant. A. We sketch the organization of the splice variant RA of the *CG13397* in: a transposon insertion mutant (M1) and corresponding control strain (C0) as well as three independent control genotypes obtained by precise excision of the mutagen transposon (C1, C2, C3) and a deletion mutant obtained by imprecise excision of the mutagen transposon (M2). Please note that C0 and M1 are the same genotypes as used in the respective panel of Fig 4 (see S7 Table for full genotypes). Boxes represent exons (black and white filling for coding and non-coding regions, respectively), whereas the fat grey arrows represent the transposon. Arrows 1, 2 and 3 indicate the binding sites for the PCR primers. Expected amplification products are marked by dashed lines. Please note that in M2, in addition to a 574 nt-long deletion, a 79 nt-long residue of the transposon remained inserted. Expected fragments were obtained in each genotype using either primers 1 and 2, or 1 and 3 in single-fly PCR experiments. B. The deletion mutant (M2) performed worse than each of the controls (C1, C2, C3) in shock avoidance. *: *P*< 0.05/ 3. Sample sizes are N = 28 for each genotype. Box plots are as explained in Fig 1B.

For independent testing, we chose 14 candidate genes which were associated with shock avoidance in terms of expression level (marked in S4 Table). In making this selection, the main restricting criterion was the availability of appropriate homozygous-viable transposon insertion mutants [25–27] (see S7 Table for full genotypes), rather than known function or the gene featuring in the interaction network in Fig 3. For 6 of the 14 probed candidate genes, we found a significant difference in scores between the respective mutant and the control (Fig 4: Mann-Whitney U-tests: FDR< 0.05; see S7 Table for full statistical reports). For the genes *CG3711*, *rad50* and *CG15107*, the respective mRNA levels were also reduced in the mutant as revealed by real time quantitative RT-PCR (S2 Fig, S7 Table). This agreed well with the behavioural impairment in the mutants, because these three genes were associated with shock avoidance such that the higher their expression levels were, the stronger the behavioural scores were across the inbred strains. For the other three genes we found no evidence for a change in the respective mRNA level due to the transposon insertion (S2 Fig; S7 Table). For 8 of the 14 probed candidate genes, the transposon insertions formally had no effect on the shock avoidance scores (Fig 4: Mann-Whitney U-tests: FDR≥ 0.05; see S7 Table for full statistical reports), although, for *CG13397* the mutants tended to avoid shock less strongly than the controls (Fig 4: Mann-Whitney U-test: FDR = 0.052; see S7 Table for full statistical reports). For this case, we re-mobilized the mutagen transposon, obtaining three fly strains where the respective locus was restored to wild-type (Fig 5A: Controls C1, C2, C3) and one strain with a deletion in the *CG13397* gene (Fig 5A: Deletion mutant M2). The deletion mutant indeed turned out to perform worse than each of the controls (Fig 5B: Mann-Whitney U-tests: U = 182.00, 243.50 and 174.00 in comparisons of M2 to C1, C2, C3; *P*< 0.05/ 3 and N = 28 in each case).

**Fig 6:**
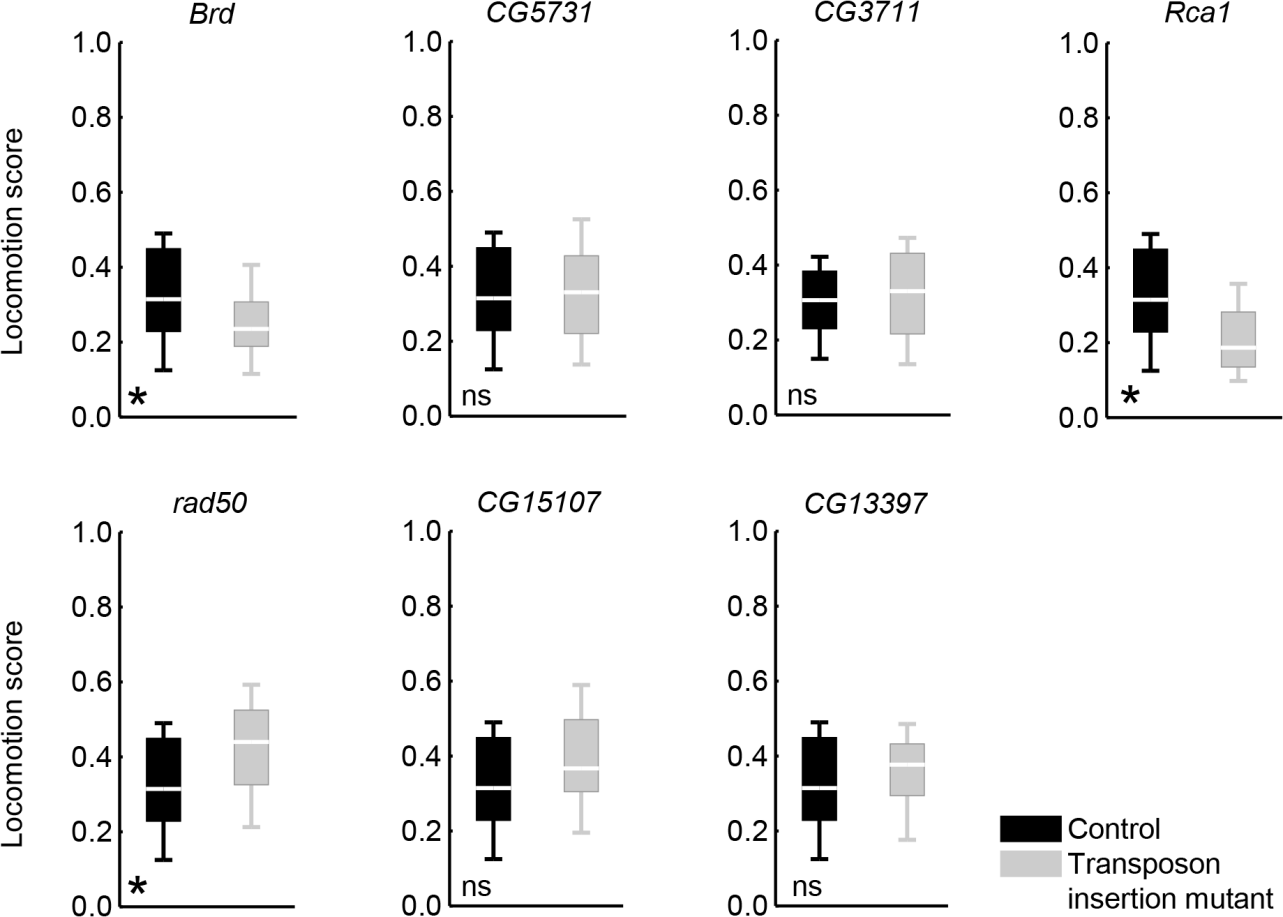
Effects of validated shock avoidance candidate genes on locomotion. Each panel shows, for the indicated gene, the locomotion scores of a respective transposon insertion mutant *vs*. those of the corresponding control. Please note that the genotypes are identical to those used in the respective panels of Fig 4 (see S7 Table for full genotypes). We used a locomotion assay that mimicked the shock avoidance assay except for the presentation of shock. Thus, using the setup and temporal schedule depicted in Fig 1A, at 6:00 min, instead of delivering shock pulses, the setup was vigorously shaken to force flies to the end of one maze-arm. The setup was then immediately put back to its horizontal position, letting the flies disperse towards the opposite arm in the absence of shock. The maze was sealed 25 s later and the locomotion score was calculated to reflect the ratio of flies that had travelled more than a shock tube-length in the given time. In 3 out of 7 cases, locomotion scores significantly differed between the genotypes. *: FDR< 0.05, ns: FDR≥ 0.05. Sample sizes are given in S7 Table. Box plots are as explained in Fig 1B.

Thus, we were able to obtain an initial validation of the roles of 7 of our candidate genes in shock avoidance, out of 14 tested. These roles may be executed at any level from peripheral shock sensation, down to the locomotor function necessary for the avoidance of shock. With this in mind, we designed a ‘locomotion assay’, which mimicked the shock avoidance assay except for the application of shock. Using this assay, we tested for the effects of the 7 validated shock avoidance candidate genes (Figs 4 and 5) on the type of locomotor function that is required in the shock avoidance assay but is *per se* irrelevant for the sensation of shock. In 4 out of 7 cases, the respective transposon insertion had no effect on the locomotion scores (Fig 6: Mann-Whitney U-tests: FDR≥ 0.05; see S7 Table for full statistical reports), suggesting that the respective impairments in shock avoidance (Fig 4) are likely to be due to changes in shock sensation, rather than in locomotor function. By contrast, for the *Rca1* gene, the respective transposon insertion lowered locomotion scores as compared to controls (Fig 6: Mann-Whitney U-test: FDR = 0.004; see S7 Table for full statistical reports); the respective impairment in shock avoidance (Fig 4) may well be secondary to this effect, rather than being due to an effect on shock sensation. For the gene *Brd*, mutants had decreased locomotion scores as compared to controls (Fig 6: Mann-Whitney U-test: FDR = 0.036; see S7 Table for full statistical reports), contrasting with their improved shock avoidance (Fig 4). As regards the gene *rad50*, the mutants had higher locomotion scores than the controls (Fig 6: Mann-Whitney U-tests: FDR = 0.023; see S7 Table for full statistical reports), contrasting with their impaired shock avoidance (Fig 4). Although for *Brd* and *rad50*, the effects of the respective transposon insertions on shock avoidance and on locomotion scores are in opposite directions, we cannot exclude the possibility that these effects are related. These two genes as well as *Rca1* should therefore not be taken as shock sensation genes. Nevertheless, it may interesting to note that *Brd*, *rad50* and *Rca1* are implicated in the development of mechanosensory bristles [28–30], which were suggested to be relevant for shock avoidance by our gene network analysis (Fig 3 and S6 Table). In addition, *Rca1* and *CG3711* have been discovered to be nociception-relevant in an RNAi-based screen [20]. Encouraged by this, we probed whether *CG3711* may be relevant for shock-reinforced associative learning, too. We trained *CG3711*-mutant as well as corresponding control flies *en masse* with a single paired presentation of an odour and pulses of electric shock. Indeed, upon such training, mutant flies showed significantly weaker conditioned avoidance of the odour as compared to the controls (S3 Fig; see the legend for methodological details).

## Materials and Methods

### Flies

Flies were kept in mass culture on standard cornmeal-molasses food [31] at 60–70% relative humidity and 25°C temperature under a 12: 12 h light: dark cycle. 1–3-day-old adults were collected in fresh food bottles and kept under the mentioned culture conditions except at 18°C temperature, for 1–3 days, so that they were 2–4 days old on the experimental day.

For the association analyses, we used 38 inbred fruit fly *Drosophila melanogaster* strains from the Drosophila Genetic Reference Panel collection (all available from the Bloomington Stock Center). These had been generated by full-sib inbreeding of iso-female strains from Raleigh, North Carolina, USA for more than 20 generations [12,13]. For independent testing of candidate genes, we used appropriate transposon insertion mutants of the Berkeley Drosophila Genome Project [25–27] along with the corresponding controls, i.e., the *white* ^*1118*^-mutant strains used for generating the respective transposon insertion mutant-collections (see S7 Table for full genotypes). In addition, the transposon inserted in the gene *CG13397* was remobilized by crossing the respective mutant strain to a transposase-positive strain (FlyBase strain ID: FBst0003612; available from the Bloomington Stock Center). This resulted in three independent cases of precise excision of the transposon, resulting in a wild-type locus (Fig 5A: Controls C1, C2, C3); as well as one case of 574 bp deletion covering parts of the first and second exon of *CG13397* (2L: 8411078 to 2L: 8411653) (Fig 5A: Deletion mutant M2), as revealed by single-fly PCR analysis (see S1 Text for details) and sequencing. Please note that in the deletion mutant, a 79 bp-long residue of the transposon remained inserted (Fig 5A).

### Electric shock avoidance assay

All experiments took place at 21–23°C temperature, 60–70% relative humidity under bright fluorescent light. Flies were tested in groups of ~ 50. At 0:00 min (Fig 1A), flies were gently introduced into a tube of 9 cm length and 1.5 cm inner diameter, coated inside with a copper wire coil and perforated at one end. This ‘shock tube’ (coloured yellow in Fig 1A) was attached to the experimental setup. At 1:00 min, with vigorous shaking, flies were transferred to a cylindrical compartment of 1.5 cm-diameter and 1 cm-length (coloured orange in Fig 1A). At 4:00 min, this ‘mid-compartment’ was gently moved to the meeting point of a maze with two shock tubes as two arms. At 6:00 min, one of the arms was electrified with 4 pulses of ~ 100 V direct current; each pulse lasted 1.2 s and had an onset-to-onset interval of 5 s to the next pulse. 10 s after the last shock-pulse, the arms of the maze were sealed and flies of each gender in each arm were counted to calculate a ‘unisex’, a ‘female’ or a ‘male’ score as

$$\text{Shock avoidance score}=\left({\#}_{\text{Shock}}–{\#}_{\text{No shock}}\right)\cdot 100\;/\;{\#}_{\text{Total}}$$ (1)

where # denotes the respective number of flies. The resulting values ranged between -100 and 100, more negative values meaning stronger avoidance of shock. The side of the electrified maze arm with respect to the setup was switched in alternating experiments to cancel out possible bias. The data from these two conditions were then pooled, except in Fig 5B, where they were pair-wise averaged. The experimental setup had four positions for processing four groups in parallel. The testing of each genotype at each position was balanced.

### Locomotion assay

We designed a locomotion assay that was directly comparable to the above-described shock avoidance assay in that it employed the same setup and had the same temporal flow. Following Fig 1A, flies were introduced into a shock tube (coloured yellow in Fig 1A) at 0:00 min, which was then attached to the setup. At 1:00 min, they were transferred to the mid-compartment (coloured orange in Fig 1A) and at 4:00 min they gained access to the maze with two shock tubes as two arms. At 6:00 min, instead of delivering shock pulses, we vigorously shook the setup such that the flies fell to the end of one arm of the maze (i.e. ‘Start arm’). Then, we immediately put the setup back to its horizontal position and let the flies disperse back towards the ‘Opposite arm’ for 25 s, in the absence of shock, before sealing the maze and counting the flies in each arm and in the mid-compartment. We calculated the

$$\text{Locomotion score}=1-{\#}_{\text{Start}–\text{arm}}/\;\left({\#}_{\text{Start arm}}+\;{\#}_{\text{Opposite arm}}+\;{\#}_{\text{Mid}–\text{compartment}}\right)$$ (2)

where # denoted the respective number of flies. Thus, the locomotion score reflects the ratio of flies that travelled more than a shock tube-length in the given time, following mechanical disturbance, rather than in response to electric shock.

### Statistical analysis of behavioural scores

Behavioural scores were analyzed using Statistica version 11.0 (StatSoft, Hamburg, Germany) and R version 2.15.1 (www.r-project.org) on a PC. We used non-parametric statistics: Kruskal-Wallis tests for global and Mann-Whitney U-tests for pair-wise comparisons. In Figs 4 and 6, as respectively 14 and 7 Mann-Whitney U-tests were performed in parallel, we calculated the Benjamini Hochberg False Discovery Rates (FDR) [32], e.g., a significance threshold of FDR< 0.05 indicated that up to 5% of the cases that were taken to be significant were expected to be false positives. In Fig 5B, as only 3 parallel comparisons were being made, we opted for a more stringent correction, dividing the critical *P*-value by the number of tests (i.e. Bonferroni correction).

### Gene expression level—shock avoidance association analysis

R version 2.15.1 (www.r-project.org) was used for these analyses. Raw Affymetrix GeneChip Drosophila Genome 2.0 expression microarray data [12] for the 38 inbred strains were downloaded from www.ebi.ac.uk/arrayexpress (accession number EMEXPE-MEXP-1594) using the R Affy package [33]. The raw data covered 18 769 probe-sets and included four expression arrays per strain, two for each gender. For the strain 399 the data from one ‘female’ sample was excluded from analysis, because the distribution of expression levels across the probe-sets rather resembled the typical ‘male’ distribution, deduced from all male samples. For all remaining data, perfect match probe intensity values were pre-processed with variance stabilization normalization (VSN) and summarized with the median polish method to obtain probe-set expression levels using the command ‘vsnrma’ with the default parameter settings [34]. For each probe-set, expression levels were averaged across samples from each strain to obtain mean unisex expression levels. These were then tested for effects on the median unisex shock avoidance scores (Fig 1B), using the following linear model:

Median shock avoidance score~β0+β1·Mean expression level (3)

β0 was the intercept and β1 the estimate for the effect of the mean expression level. β1 was compared to zero with a two-tailed t-test (d.f. = 356). The probe-sets with a *P*< 0.05 were considered to be associated with shock avoidance (see S1 Table for full statistical reports) and annotated according to Affymetrix documentation (www.affymetrix.com) and the FlyBase [23] (www.flybase.org). To determine the candidate genes, we excluded the probe-sets with ‘_x_’ or ‘_s_’ qualifiers in their probe-set Affymetrix IDs, as these contain one or more probes that hybridize with products of different genes. Those genes for which at least one corresponding probe-set fulfilled the statistical criterion for association were considered to be candidates (S4 Table).

### Single nucleotide polymorphism (SNP)—shock avoidance association analysis

The Illumina and 454 SNP calls of the 38 inbred strains [13] were downloaded from http://dgrp.gnets.ncsu.edu/data/. We pre-selected bi-allelic, homo-/ hemizygous single nucleotide polymorphisms (SNPs) with minor allele frequency (MAF)> 0.1 (calculated over the 38 strains) and call-rate> 0.7. For each such SNP, we tested for an effect on the shock avoidance scores using the following linear model:

Median shock avoidance score~β0+β1·Allele (4)

The minor and major alleles took the values 2 and 0, respectively. β0 was the intercept, whereas β1 was the estimate for the effect of the allele. β1 was subjected to a two-tailed t-test comparing it to zero. With respect to autosomal SNPs, this analysis was done using the ‘unisex’ shock avoidance scores (Fig 1B). The cases with *P*< 0.0005 were considered to be suggestive associations (see S2 Table for full statistical reports). With respect to the sex-chromosome SNPs, we did this analysis separately for each sex, using the sex-specific shock avoidance scores (S1 Fig). Considering the hemizygous state of the males, the male β1 values were multiplied by two. Those SNPs that had *P*< 0.0005 in at least one sex were taken as associated with shock avoidance (see S3 Table for full statistical reports). All shock avoidance-associated SNPs were annotated according to *Drosophila melanogaster* reference genome version 5.35 and the FlyBase [23] (www.flybase.org). For defining the candidate genes, those SNPs that were annotated to multiple genes were excluded and those genes that had at least one SNP fulfilling the statistical criterion for association were taken as candidates (S4 Table).

### Enrichment of gene ontology (GO) terms

To probe for the enrichment of GO terms for Biological Process, Cellular Compartment and Molecular Function, we analyzed our list of 514 candidate shock avoidance genes (S4 Table) against the background of the *Drosophila melanogaster* genome, using the Functional Annotation Clustering tool of DAVID 6.7 (http://david.abcc.ncifcrf.gov/home.jsp) [35,36] with default settings. S5 Table lists the annotation clusters with an Enrichment Score > 1.

### Gene interaction network analysis

This analysis followed up on the gene expression level—shock avoidance associations. On the one hand, for each of the 18 769 probe-sets considered for association, we obtained gene FlyBase IDs, using the R Bioconductor package drosophila2.db (www.bioconductor.org) and the Batch Processing Tool of FlyBase [23] (www.flybase.org). On the other hand, the protein—protein interaction data were downloaded from FlyBase (ftp://ftp.flybase.net/releases/current/precomputed_files/genes/physical_interactions_fb_2014_05.tsv.gz). From the intersection of these two datasets a network of 5 280 genes and 63 796 pair-wise interactions was derived. For each gene in this network, the *P* value for the association between the expression level and the shock avoidance scores was converted into a network node score based on the negative decadic logarithm of the *P* value. Subsequently, these scores were adjusted using a *P* value cut-off of 0.025, such that only *P* values smaller than this threshold were positively scored, while the remaining non-significant *P* values obtained negative scores. Using an integer linear programming formulation, the optimally scoring sub-module was calculated exactly [24], resulting in a specific, smaller shock avoidance-associated network. This network was visualized using the routines in the BioNet framework [37]. S6 Table lists all network genes along with annotation as well as relevant statistics of their association with shock avoidance.

## Supporting Information

S1 Fig Sex-specific shock avoidance scores of 38 inbred strains. The data from Fig 1B are separately plotted for each sex. Both female (red) and male (blue) shock avoidance scores significantly varied across strains (Kruskal-Wallis tests: H = 145.10 and 107.60, respectively; d.f. = 37, *P*< 0.0001 in each case; N for females = 32, 16, 22, 23, 23, 16, 16, 24, 25, 28, 15, 24, 26, 34, 16, 32, 24, 22, 18, 18, 21, 31, 16, 14, 24, 14, 28, 18, 22, 15, 30, 16, 20, 24, 16, 18, 32, 24; N for males = 32, 15, 21, 24, 24, 16, 16, 23, 26, 27, 16, 23, 25, 33, 16, 32, 23, 21, 18, 18, 22, 32, 15, 13, 24, 15, 28, 18, 21, 14, 29, 16, 20, 24, 15, 19, 28, 22). The small differences in sample sizes across sexes arose because the shock avoidance scores calculated on the basis of less than 5 individual flies were excluded from analysis. Box plots as in Fig 1B.
(TIF)

S2 Fig Real-time quantitative RT-PCR analysis of the transposon insertion mutants in Fig 4. Fig 4 compares for 14 selected candidate genes, the respective transposon insertion mutants with the corresponding controls in terms of shock avoidance. S2 Fig. in turn presents, for 12 of these cases, the respective mRNA levels, as measured by real-time quantitative RT-PCR (see S1 Text for details). In **A**, each panel shows for the indicated gene the gender-specific Delta CT values of mutant *vs*. control in a scatter plot. For example, as in the case of *CG15107* females, if the control had a median Delta CT of ~ 7, while the mutant had ~ 10, this indicated that the mutant mRNA level was ~ 2^(7–10)^ = 0.125^th^ of the control. In B, these fold change values (also see S7 Table) are plotted on a logarithmic axis, such that value one would indicate that the respective mRNA-level in the mutant were equal to those in the control; whereas values below and above one would indicate decreased and increased mRNA levels in the mutant, respectively. Thus, the mRNA levels of *CG3711*, *rad50* and *CG15107* were clearly reduced in the respective mutants, accompanying the impairment in shock avoidance (Fig 4). For *Rca1*, the mutants’ defective shock avoidance (Fig 4) was not paralleled by a decrease in the mRNA level. As regards *Brd* and *CG5731*, for which we found an effect of the transposon insertion on shock avoidance (Fig 4), the quantification of mRNA turned out to be unfeasible, probably due to low expression levels (modENCODE Temporal Expression Data [www.flybase.org]) [1]. In addition, for *Tsp42Ei*, *CG16865* and *CG3290*, reductions in the respective mRNA levels were found in the mutants, although shock avoidance was comparable to the controls (Fig 4). In the remaining cases, the transposon insertion seemed neither to decrease the respective mRNA levels, nor to affect the shock avoidance scores (Fig 4).
(TIF)

S3 Fig Role of *CG3711* in shock-reinforced olfactory associative learning. A. For the shock-reinforced olfactory learning assay, flies entered the setup at 0:00 min and were presented with a control odour from 4:00 min on for 15 s. A trained odour was in turn applied from 7:15 min on for 15 s, immediately followed by electric shock (100 V direct current, 4 pulses each 1.2 s-long and followed by the next pulse with an onset-to-onset interval of 5 s). At 12:00 min, flies were transferred to food vials to rest until they were re-introduced into the setup at 28:00 min. At 33:00 min, they were brought to the mid-point of a maze with two arms scented with either odour and were allowed to choose for 2 min. At the end of the choice, the maze-arms were sealed and the flies were counted to calculate an odour preference score as PREF = (#_Trained odour_—#_Control odour_) 100 / #_Total_, where # indicates the number of flies in the respective maze-arm. Two subgroups of flies were always trained in parallel, switching the roles of two chemicals as control and trained odour. We used the odours 3-octanol (OCT, Merck Schuchardt, Hohenbrunn, Germany, CAS: 589-98-0, applied undiluted into Teflon cups of 14 mm diameter) and benzaldehyde (BA, Merck Schuchardt, Hohenbrunn, Germany, CAS: 100-52-7, applied undiluted into Teflon cups of 5 mm diameter). A learning index was calculated based on the preferences of these two groups, in order to cancel out non-associative effects. Learning index = (PREF_BA-Shock_ + PREF_OCT-Shock_) / 2, where the subscripts of PREF indicate the respective odour-shock contingency. Thus, negative learning indices indicated conditioned avoidance from the trained odour, whereas positive values indicated conditioned approach. B. A transposon insertion mutant of *CG3711* performed worse than its corresponding control not only in shock avoidance (Fig 4), but also in shock-reinforced olfactory learning (Mann-Whitney U-test: U = 1221.00, *P*< 0.05, N = 61, 60 for the control and mutant flies, respectively). Box plots as in Fig 1B.
(TIF)

S1 Table Gene expression level associations. For each probe-set, we tested for a linear regression between the mean expression levels and the median unisex shock avoidance scores. β1 is the respective estimate for the effect of the expression level on shock avoidance. Negative β1 values indicate that the higher the expression level was, the stronger the shock avoidance was; positive β1 values reflect the converse, i.e., the higher the expression level, the weaker the shock avoidance. The t and *P* values refer to the results of a two-tailed t-test comparing β1 to zero. We list probe-sets with *P* < 0.05. Annotations are based on Affymetrix documentation (www.affymetrix.com) and the FlyBase (www.flybase.org) [1].
(XLSX)

S2 Table Autosomal single nucleotide polymorphism (SNP) associations. For each bi-allelic autosomal SNP with a favourable minor allele frequency and call rate, we tested for a linear regression between the allele type and the median unisex shock avoidance scores. β1 is the respective estimate for the effect of allele type on shock avoidance. The t and *P* values refer to the results of a two-tailed t-test comparing β1 to zero. We list SNPs with *P*< 0.0005. Annotations are based on *Drosophila melanogaster* reference genome version 5.35 and the FlyBase [1].
(XLSX)

S3 Table X-chromosome-linked single nucleotide polymorphism (SNP) associations. For each bi-allelic X-chromosome-linked SNP with a favourable minor allele frequency and call rate, we tested for a linear regression between the allele type and either the female or the male median shock avoidance scores. β1, t, and P are as explained for S2 Table. We list SNPs with *P*< 0.0005 in at least one gender. Annotations are based on *Drosophila melanogaster* reference genome version 5.35 and the FlyBase [1].
(XLSX)

S4 Table Candidate shock avoidance genes. We list the candidate shock avoidance genes revealed by expression level- and/ or SNP-associations (from S1–S3 Tables). In constructing this list, the probe-sets and SNPs with ambiguous annotation were excluded. Independent validation refers to the results presented in Figs 4 and 5 as well as S7 Table.
(XLSX)

S5 Table Functional annotation analysis. Based on DAVID 6.7 (http://david.abcc.ncifcrf.gov/home.jsp) [14,15], we list the functional annotation clusters that were enriched (Enrichment Score > 1) amongst our 514 candidate shock avoidance genes as compared to the *Drosophila melanogaster* genome. (XLSX)

S6 Table Network genes. By superimposing the results of our gene expression level—shock avoidance analyses on the existing protein-protein interaction network, we obtained a shock avoidance-relevant network of 38 genes, which are listed along with the statistics of their association with shock avoidance (see the legend of S1 Table for details), as well as their relevance for bristles.
(XLSX)

S7 Table Independent validation of candidate genes. For 14 candidate genes from S4 Table, we compared appropriate transposon insertion mutants to controls in terms of shock avoidance and the level of the respective mRNAs as measured by real-time quantitative RT-PCR. For 7 of these genes, locomotion assays were also run. Here, the results of these analyses are documented in detail.
(XLSX)

S1 Text Supplemental methods and references. Detailed methodology for PCR and real-time quantitative PCR are given, along with a reference list for these as well as the Supplemental Tables and Supplemental Figures.
(DOCX)
