# Supplementary figures and images for: Genome-Wide Association Analyses Point to Candidate Genes for Electric Shock Avoidance in Drosophila melanogaster
Source: PLoS One. 2015 May 18;10(5):e0126986. doi: 10.1371/journal.pone.0126986 (PMC4436303; doi:10.1371/journal.pone.0126986)

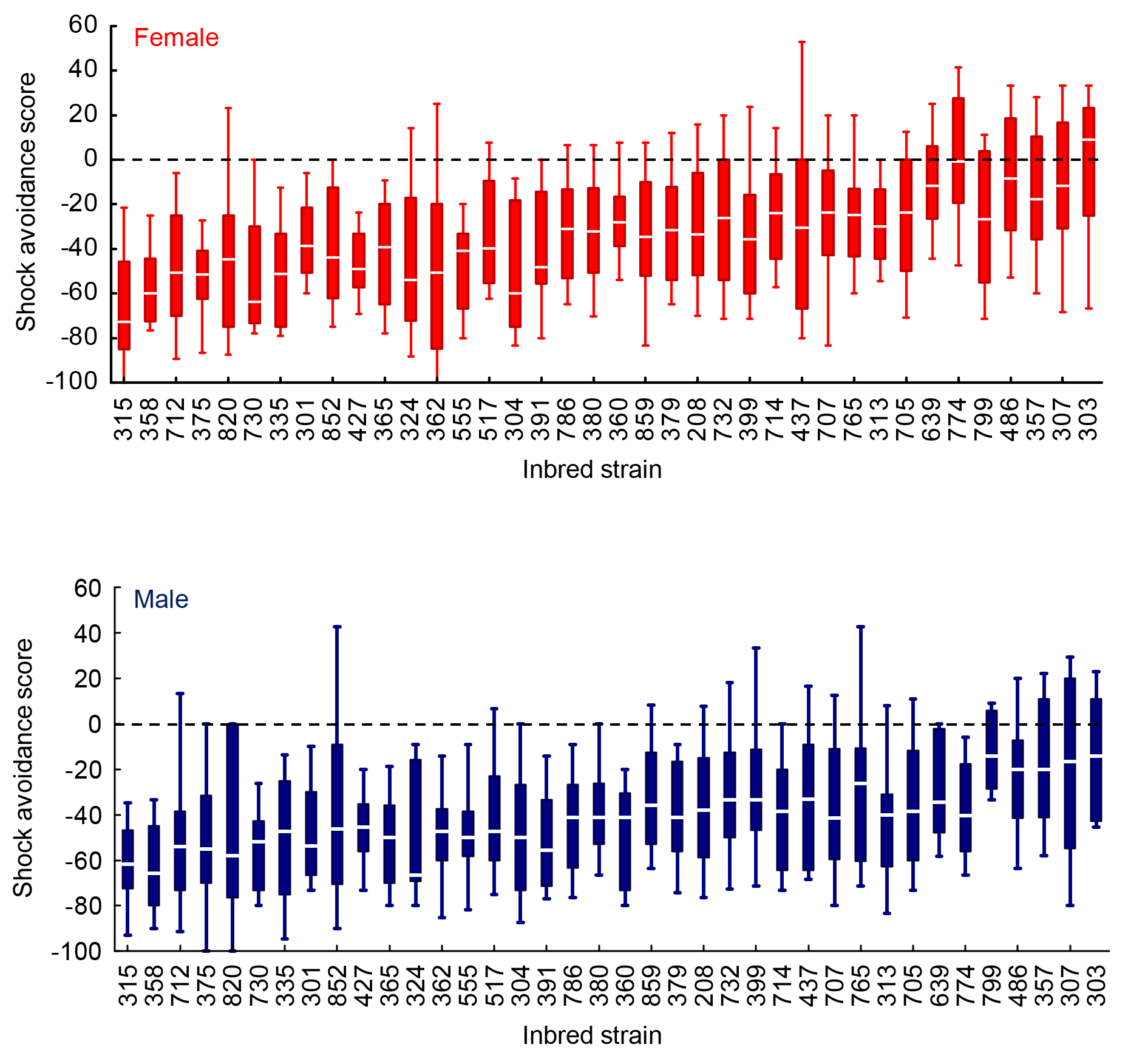

Supplement: S1 Fig — The data from Fig 1B are separately plotted for each sex. Both female (red) and male (blue) shock avoidance scores significantly varied across strains (Kruskal-Wallis tests: H = 145.10 and 107.60, respectively; d.f. = 37, P< 0.0001 in each case; N for females = 32, 16, 22, 23, 23, 16, 16, 24, 25, 28, 15, 24, 26, 34, 16, 32, 24, 22, 18, 18, 21, 31, 16, 14, 24, 14, 28, 18, 22, 15, 30, 16, 20, 24, 16, 18, 32, 24; N for males = 32, 15, 21, 24, 24, 16, 16, 23, 26, 27, 16, 23, 25, 33, 16, 32, 23, 21, 18, 18, 22, 32, 15, 13, 24, 15, 28, 18, 21, 14, 29, 16, 20, 24, 15, 19, 28, 22). The small differences in sample sizes across sexes arose because the shock avoidance scores calculated on the basis of less than 5 individual flies were excluded from analysis. Box plots as in Fig 1B. (TIF) [file pone.0126986.s001.tif]

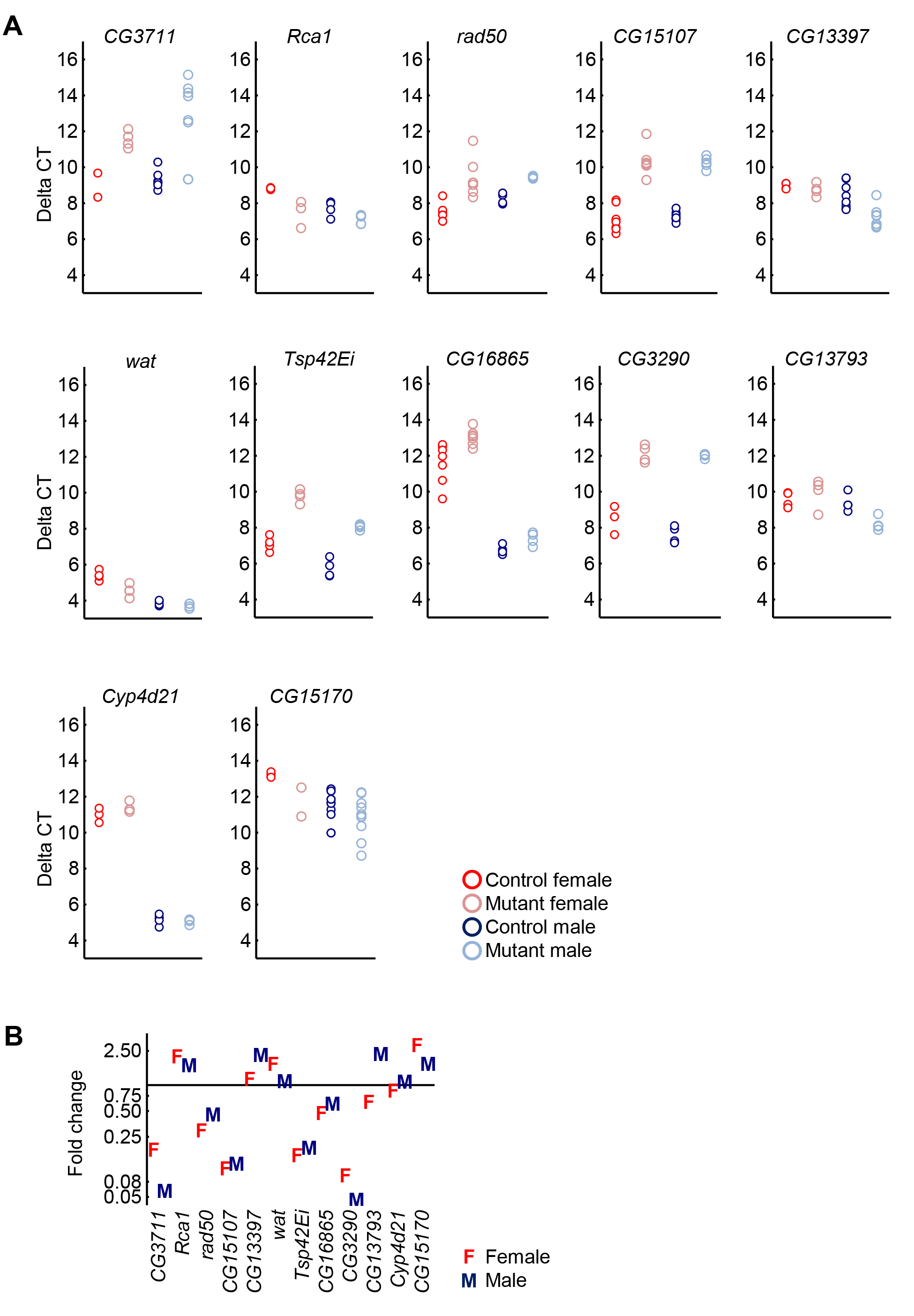

Supplement: S2 Fig — Fig 4 compares for 14 selected candidate genes, the respective transposon insertion mutants with the corresponding controls in terms of shock avoidance. S2 Fig. in turn presents, for 12 of these cases, the respective mRNA levels, as measured by real-time quantitative RT-PCR (see S1 Text for details). In A, each panel shows for the indicated gene the gender-specific Delta CT values of mutant vs. control in a scatter plot. For example, as in the case of CG15107 females, if the control had a median Delta CT of ~ 7, while the mutant had ~ 10, this indicated that the mutant mRNA level was ~ 2(7–10) = 0.125th of the control. In B, these fold change values (also see S7 Table) are plotted on a logarithmic axis, such that value one would indicate that the respective mRNA-level in the mutant were equal to those in the control; whereas values below and above one would indicate decreased and increased mRNA levels in the mutant, respectively. Thus, the mRNA levels of CG3711, rad50 and CG15107 were clearly reduced in the respective mutants, accompanying the impairment in shock avoidance (Fig 4). For Rca1, the mutants’ defective shock avoidance (Fig 4) was not paralleled by a decrease in the mRNA level. As regards Brd and CG5731, for which we found an effect of the transposon insertion on shock avoidance (Fig 4), the quantification of mRNA turned out to be unfeasible, probably due to low expression levels (modENCODE Temporal Expression Data [www.flybase.org]) [1]. In addition, for Tsp42Ei, CG16865 and CG3290, reductions in the respective mRNA levels were found in the mutants, although shock avoidance was comparable to the controls (Fig 4). In the remaining cases, the transposon insertion seemed neither to decrease the respective mRNA levels, nor to affect the shock avoidance scores (Fig 4). (TIF) [file pone.0126986.s002.tif]

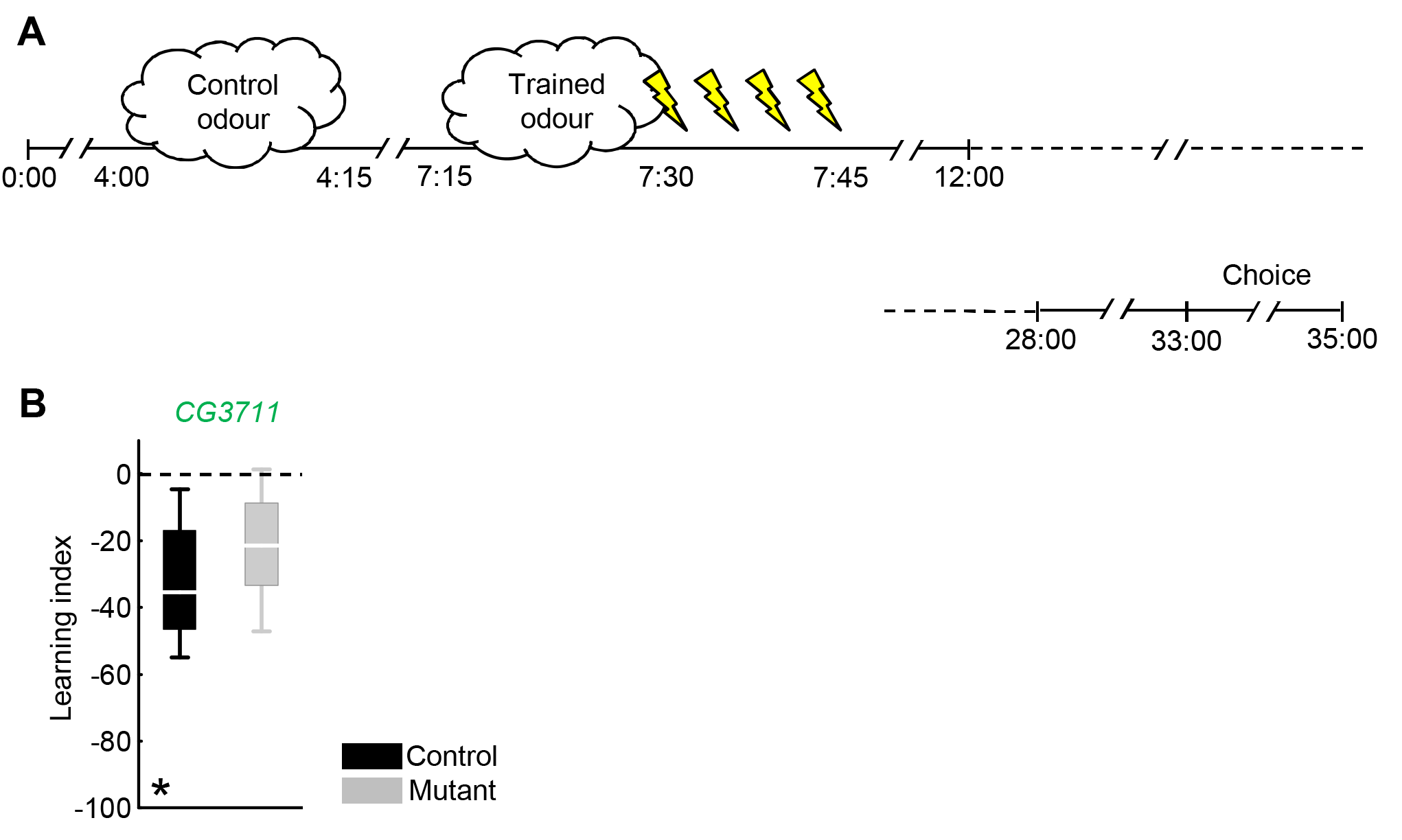

Supplement: S3 Fig — A. For the shock-reinforced olfactory learning assay, flies entered the setup at 0:00 min and were presented with a control odour from 4:00 min on for 15 s. A trained odour was in turn applied from 7:15 min on for 15 s, immediately followed by electric shock (100 V direct current, 4 pulses each 1.2 s-long and followed by the next pulse with an onset-to-onset interval of 5 s). At 12:00 min, flies were transferred to food vials to rest until they were re-introduced into the setup at 28:00 min. At 33:00 min, they were brought to the mid-point of a maze with two arms scented with either odour and were allowed to choose for 2 min. At the end of the choice, the maze-arms were sealed and the flies were counted to calculate an odour preference score as PREF = (#Trained odour—#Control odour) 100 / #Total, where # indicates the number of flies in the respective maze-arm. Two subgroups of flies were always trained in parallel, switching the roles of two chemicals as control and trained odour. We used the odours 3-octanol (OCT, Merck Schuchardt, Hohenbrunn, Germany, CAS: 589-98-0, applied undiluted into Teflon cups of 14 mm diameter) and benzaldehyde (BA, Merck Schuchardt, Hohenbrunn, Germany, CAS: 100-52-7, applied undiluted into Teflon cups of 5 mm diameter). A learning index was calculated based on the preferences of these two groups, in order to cancel out non-associative effects. Learning index = (PREFBA-Shock + PREFOCT-Shock) / 2, where the subscripts of PREF indicate the respective odour-shock contingency. Thus, negative learning indices indicated conditioned avoidance from the trained odour, whereas positive values indicated conditioned approach. B. A transposon insertion mutant of CG3711 performed worse than its corresponding control not only in shock avoidance (Fig 4), but also in shock-reinforced olfactory learning (Mann-Whitney U-test: U = 1221.00, P< 0.05, N = 61, 60 for the control and mutant flies, respectively). Box plots as in Fig 1B. (TIF) [file pone.0126986.s003.tif]
