# Supplementary material for: Genome-Wide Association Analyses Point to Candidate Genes for Electric Shock Avoidance in Drosophila melanogaster
Source: PLoS One. 2015 May 18;10(5):e0126986. doi: 10.1371/journal.pone.0126986 (PMC4436303; doi:10.1371/journal.pone.0126986)
Supplement: S1 Text — Detailed methodology for PCR and real-time quantitative PCR are given, along with a reference list for these as well as the Supplemental Tables and Supplemental Figures. (DOCX) [file pone.0126986.s011.docx]

**S1 Text: Supplemental text with methods and references**

**Supplemental methods**

*Single-fly PCR*

We followed the procedure of [17], using material from individual flies. The binding sites were upstream of the deletion for primer 1 (5’-CAAATGGCCTGAGGATCAAT-3’), downstream of the deletion for primer 2 (5’-GATGATCCCTCGCATTTCAG-3’), and within the transposon for primer 3 (5’-CGACACTCAGAATACTATTCC-3’).

*RNA extraction, reverse transcription and real-time quantitative RT-PCR*

Flies aged 1- 3 days after eclosion were collected in food vials and kept at 18 °C overnight, so that on the day of sample preparation they were 2- 4 days old. Flies were transferred into pre-frozen empty vials, which were then quickly put into liquid nitrogen. Frozen flies were sorted on a Petri dish on dry ice into groups of 10 males or 10 females. Each such group was placed back into a falcon tube and put back into liquid nitrogen to be stored at -80 °C until RNA extraction. For RNA extraction, we used the Trizol Reagent following the manufacturer’s protocol (Sigma Aldrich, Taufkirchen, Germany; product number T9424-200ML). The resulting RNA content was measured by spectrophotometry on a Nanodrop2000 (PeqLab Biotechnology GmbH, Erlangen, Germany), and each RNA sample was accordingly diluted with RNAse free water to a final concentration of 1 µg/ µl. 10 µl of each such sample was then used for reverse transcription with Superscript III and random primers (Life technologies, Invitrogen, Darmstadt, Germany; product numbers 18080-044 and 48190-011), following the manufacturer’s protocol. The resulting cDNA samples were stored at -20 °C until real-time quantitative PCR, which was performed on a 7500 Fast Real-Time PCR System (Applied Biosystems, Darmstadt, Germany) following the manufacturer’s protocol, thus using 8µl of cDNA sample, 2µl of a primer-mix (5 pmol/ µl per primer) and 10µl FastSybrGreen Master Mix (Applied Biosystems, Darmstadt, Germany; product number 4385614) to reach a total of 20 µl reaction volume. For each gene of interest and the housekeeping gene *rp49*, we designed 20 b forward and reverse oligonucleotide primers, such that the amplified product size ranged 70- 150 bp (see the table below for primer sequences). For primer-design, the software Primer3 version 4.0 ( <http://frodo.wi.mit.edu/>, [18]) together with the *Drosophila* mis-priming library was used. The oligonucleotides were synthesized by Metabion (Martinsried, Germany).

We analyzed with real-time quantitative PCR 2-12 independent cDNA samples for each genotype and sex (S7 Table). Each sample that was included in a given run was loaded twice, once to amplify a fragment of the cDNA of interest and once to amplify a fragment of *rp49* cDNA (<http://www.roche-applied-science.com/sis/rtpcr/upl/index>). For each case, a CT value was calculated as the number of cycles to reach a particular amount of amplified product, characterized by a fluorescence intensity threshold of ~ 0.05, which was in the linear phase of the cycle number – fluorescence intensity curves. Then, for each sample, a Delta CT was calculated by subtracting the CT value of the *rp49*-amplification from the CT value of the gene of interest-amplification. These Delta CT values were then plotted comparatively between mutants and controls for each sex (S2A Fig.). To assess the fold changes in S7 Table, we calculated for each genotype and sex the Median Delta CT values. If, e.g, the Median Delta CT of a mutant was 4 units larger than that of the control, this implied a 2^4^ = 16- fold decrease in the level of the respective mRNA in the mutant (S2B Fig., S7 Table).

| **Targeted gene** | **Forward primer 5’🡪3’** | **Reverse primer 5’🡪3’** |
| --- | --- | --- |
| *CG3711* | GTTCCCACACTCACCTTCGT | TGCAGTCAGGGAGGTACACA |
| *Rca1* | TGGACGTACAACCAGCACAT | TCCTGACCTCGTCAAGATCC |
| *rad50* | AATTGAATCGCTGTCCATCC | ACCGGCGAGGAAAACTTTAT |
| *CG15107* | CTGGAACCATCGAGCCTTAG | GAGTTGGTGGATGAGGAGGA |
| *CG13397* | TCCAAAAGGGATTCTTCACG | GATCCCTTCAACGAACTGGA |
| *wat* | CTTTGGCCAGCTTGATGATT | CATGGAGAACGTCAACATCG |
| *Tsp42Ei* | GCCGCTGTATCCGAAAGATA | GCGCAGTAGAGTGCATCAGA |
| *CG16865* | AGCCCACAACTGGGACATAG | TAAGTGCCGTCCACTCACTG |
| *CG3290* | TTTCCACTACCAACGCATGA | ACCTTTTCACCGGAGTGATG |
| *CG13793* | GAGAACGGAAGGGACATGAA | TGCGACAATAGCTGAGGAAGT |
| *Cyp4d21* | GTGGACTTCTCGCAGAAAGG | GAATAGGTCCACGCACGTTT |
| *CG15170* | CCAAACTCCGTAACCTGCAT | TGTCGCACTTGTCACAGTCA |
| *rp49* | CGGATCGATATGCTAAGCTGT | GCGCTTGTTCGATCCGTA |

**Supplemental references**

1. Marygold SJ, Leyland PC, Seal RL, Goodman JL, Thurmond J, Strelets VB, et al. (2013) FlyBase: improvements to the bibliography. Nucleic Acids Res 41: D751-757.

2. Neely GG1, Hess A, Costigan M, Keene AC, Goulas S, Langeslag M, et al. (2010) A genome-wide Drosophila screen for heat nociception identifies α2δ3 as an evolutionarily conserved pain

gene. Cell 143: 628-638.

3. Hong S-T, Bang S, Hyun S, Kang J, Jeong K, Paik D, et al. (2008) cAMP signalling in mushroom bodies modulates temperature preference behaviour in Drosophila. Nature 454: 771-775.

4. Tan Y, Yu D, Pletting J, Davis RL (2010) Gilgamesh is required for rutabaga-independent

olfactory learning in Drosophila. Neuron 67: 810-820.

5. Quinn WG, Sziber PP, Booker R (1979) The Drosophila memory mutant amnesiac. Nature

277: 212-214.

6. Qiu Y, Davis RL (1993) Genetic dissection of the learning/memory gene dunce of

Drosophila melanogaster. Genes Dev 7: 1447-1458.

7. Gouzi JY, Moressis A, Walker JA, Apostolopoulou AA, Palmer RH, Bernards A, et. al. (2011) The receptor tyrosine kinase Alk controls neurofibromin functions in Drosophila growth and

learning. PLoS Genet 7: e1002281.

8. Bolduc FV, Bell K, Cox H, Broadie KS, Tully T (2008) Excess protein synthesis in

Drosophila fragile X mutants impairs long-term memory. Nat Neurosci 11: 1143-1145.

9. Akalal DB, Yu D, Davis RL. (2011) The long-term memory trace formed in the

Drosophila α/β mushroom body neurons is abolished in long-term memory mutants. J Neurosci 31: 5643-5647.

10. Norga KK, Gurganus MC, Dilda CL, Yamamoto A, Lyman RF, Patel PH, et al. (2003) Quantitative analysis of bristle number in Drosophila mutants identifies genes involved in neural

development. Curr Biol 13: 1388-1396.

11. Mummery-Widmer JL, Yamazaki M, Stoeger T, Novatchkova M, Bhalerao S, Chen D, et al.

(2009) Genome-wide analysis of Notch signalling in Drosophila by transgenic RNAi. Nature

458: 987-992.

12. Leviten MW, Posakony JW. (1996) Gain-of-function alleles of Bearded interfere with

alternative cell fate decisions in Drosophila adult sensory organ development. Dev Biol. 176:

264-283.

13. Ciapponi L, Cenci G, Ducau J, Flores C, Johnson-Schlitz D, Gorski MM, et al. (2004) The Drosophila Mre11/Rad50 complex is required to prevent both telomeric fusion and chromosome

breakage. Curr Biol 14: 1360-1366.

14. Huang DW, Sherman BT, Lempicki RA. (2009) Systematic and integrative analysis of large gene lists using DAVID Bioinformatics Resources. Nature Protoc 4:44-57.

15. Huang DW, Sherman BT, Lempicki RA. (2009) Bioinformatics enrichment tools: paths toward the comprehensive functional analysis of large gene lists. Nucleic Acids Res 37: 1-13.

16. Török I, Herrmann-Horle D, Kiss I, Tick G, Speer G, Schmitt R et al. (1999) Down-regulation of RpS21, a putative translation initiation factor interacting with P40, produces viable minute

imagos and larval lethality with overgrown hematopoietic organs and imaginal discs. Mol

Cell Biol 19: 2308-2321.

17. Gloor GB, Preston CR, Johnson-Schlitz DM, Nassif NA, Phillis RW, Benz WK, et al. (1993). Type I repressors of P element mobility. Genetics 135: 81–95.

18. Rozen S, Skaletsky H (2000) Primer3 on the WWW for general users and for biologist

programmers. Methods Mol Biol 132: 365-386.
